# TACCO unifies annotation transfer and decomposition of cell identities for single-cell and spatial omics

**DOI:** 10.1038/s41587-023-01657-3

**Published:** 2023-02-16

**Authors:** Simon Mages, Noa Moriel, Inbal Avraham-Davidi, Evan Murray, Jan Watter, Fei Chen, Orit Rozenblatt-Rosen, Johanna Klughammer, Aviv Regev, Mor Nitzan

**Affiliations:** 1 Klarman Cell Observatory, Broad Institute of MIT and Harvard, Cambridge, MA USA; 2 Gene Center and Department of Biochemistry, Ludwig-Maximilians-University Munich, Munich, Germany; 3 School of Computer Science and Engineering, The Hebrew University of Jerusalem, Jerusalem, Israel; 4 Broad Institute of MIT and Harvard, Cambridge, MA USA; 5 Department of Stem Cell and Regenerative Biology, Harvard University, Cambridge, MA USA; 6 Massachusetts Institute of Technology, Cambridge, MA USA; 7 Racah Institute of Physics, The Hebrew University of Jerusalem, Jerusalem, Israel; 8 Faculty of Medicine, The Hebrew University of Jerusalem, Jerusalem, Israel; 9 Present Address: Genentech, South San Francisco, CA USA

**Keywords:** Software, Data integration, Data processing, Computational platforms and environments

## Abstract

Transferring annotations of single-cell-, spatial- and multi-omics data is often challenging owing both to technical limitations, such as low spatial resolution or high dropout fraction, and to biological variations, such as continuous spectra of cell states. Based on the concept that these data are often best described as continuous mixtures of cells or molecules, we present a computational framework for the transfer of annotations to cells and their combinations (TACCO), which consists of an optimal transport model extended with different wrappers to annotate a wide variety of data. We apply TACCO to identify cell types and states, decipher spatiomolecular tissue structure at the cell and molecular level and resolve differentiation trajectories using synthetic and biological datasets. While matching or exceeding the accuracy of specialized tools for the individual tasks, TACCO reduces the computational requirements by up to an order of magnitude and scales to larger datasets (for example, considering the runtime of annotation transfer for 1 M simulated dropout observations).

## Abstract (web summary)

Annotation transfer from reference to new datasets is improved with a probabilistic approach.

## Main

Single-cell and spatial genomics methods provide high-dimensional measurements of biological systems at unprecedented scale and resolution^1,2^. One of the key challenges is attaching meaningful and interpretable representations of the experimental measurements to denote cellular features, such as types, states, cell-cycle stages or position within tissues^3,4^ to decipher cellular dynamics, communication and collective behavior. For several biological systems, annotated reference datasets exist that can be leveraged for annotating new, more complex and information-rich datasets.

**Fig. 1:**
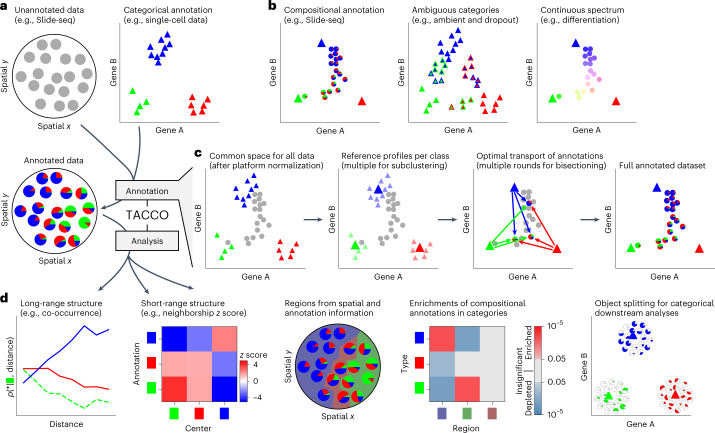
TACCO, a flexible framework for the annotation and analysis of cells and cell-like objects. **a**, TACCO generates annotations for new datasets of mixtures (top left) using an annotated single-cell reference (top right) and provides methods for downstream analysis of the resulting compositional annotations (bottom left). **b**, Compositional annotations. Illustrative embeddings of cells and cell-like objects annotated (left) for mixtures (pie charts) of idealized pure contributions (triangles); (middle) as ambiguous annotations (triangles with colored borders) for technical artifacts like high ambient contributions or dropout levels and (right) continuous annotations (circles) along biological continua. **c**, Annotation process. Far left: a labeled reference dataset (for example, scRNA-seq data and colored triangles) and a new dataset (for example, Slide-seq beads and circles) are first presented in a common high-dimensional space (for example, expression space) optionally using platform normalization to make the datasets comparable. Near left: TACCO represents the reference categories by one or multiple representative profiles (large colored triangles). Near right: TACCO uses semi-unbalanced entropic optimal transport to transfer annotations from the reference categories to the new dataset (arrows), generating compositional annotations for the new datapoints (colored pie charts). To improve the capture of subdominant contributions, this process is iterated. Far right: TACCO provides compositional annotations for the new dataset. **d**, TACCO analysis tools for compositional annotations, especially for spatial data. From left: spatial relationship analysis on long (tissue) and short (cellular neighborships) length scales; inferring spatial regions by both spatial and annotation information; enrichment of compositional annotations and splitting compositionally annotated count data into pure contributions for downstream analysis with single-cell analysis tools.

However, when the new dataset varies substantially from the reference, transferring annotations is a challenging task. A prime example is the transfer of annotations from scRNA-seq to spatial transcriptomics with supracellular spatial resolution. For instance, Slide-seq^5,6^ uses spatially scattered beads to measure the combined expression of multiple neighboring cells, possibly of different types. Transferred annotations, like cell type, thus become compositional annotations, such as cell-type fractions per bead (Fig. 1a). Similarly, for spatial data with subcellular or even single-molecule resolution^7^, compositional annotations of local neighborhoods allow segmentation-free annotation and cell assignment of single molecules. Furthermore, compositional annotations can arise not only from cell mixtures but also from ambiguity of categorical annotations (Fig. 1b). For example, technical noise, caused by dropout or contamination of ambient RNA, can mask the true identity of an individual cell. Finally, biological continua as observed in cell differentiation or regulatory cellular spectra also require probabilistic cell-type assignments (Fig. 1b).

While different decomposition or mapping methods have been developed for specific use cases such as decomposition of spatial measurements^5,8–11^, spatial single-cell mapping^12–14^ or resolving trajectories^15–18^, they are in fact conceptually similar. In particular, in each of these cases, proximity in expression space translates to higher contributions in the annotation, suggesting that it should be possible to develop a general unifying framework across these tasks.

We implement this idea in a computational framework for the transfer of annotations to cells and their combinations (TACCO). TACCO is an optimal transport-based, flexible and efficient framework for annotation and analysis of single-cell and spatial omics data. We demonstrate TACCO’s applicability in various use cases, including identification of cell types and states, analysis of spatiomolecular tissue structure at different scales and single-cell differentiation fate prediction. Finally, we show that TACCO performs favorably in terms of accuracy and computing requirements when compared to other methods.

## Results

### TACCO: a framework for transferring annotations

We developed TACCO, a fast and flexible computational decomposition framework (Fig. 1c). TACCO takes as input an unannotated dataset consisting of observations (for example, the expression profiles of Slide-seq beads) and a corresponding reference dataset with annotations in a reference representation (for example, single-cell expression profiles with cell-type annotations) and computes a compositional annotation of the unannotated observations (for example, cell-type fractions of the Slide-seq beads). Working in a common high-dimensional space (for example, gene expression space), TACCO determines these compositions using a variation of the optimal transport (OT) algorithm (Methods). At its core, OT creates a probabilistic map between the unannotated observations (for example, Slide-seq beads) and the means of the classes in the reference representation (for example, mean cell-type profiles) according to their relative similarity. This approach was previously used to map ancestor and descendant cells during differentiation^16^, recover the spatial organization of cells^13,19–21^ and associate measurements across modalities (for example, single-cell ATAC-seq and scRNA-seq)^22–24^. Through the OT framework, users can set the similarity metric, constrain or bias the marginals of the mapping and control the entropy of the annotation distributions (Methods). By default, TACCO uses Bhattacharyya coefficients as a similarity metric (Methods), which are formally equivalent to the overlaps of probability amplitudes in quantum mechanics and closely related to expectation values of measurements.

**Extended Data Fig. 1:**
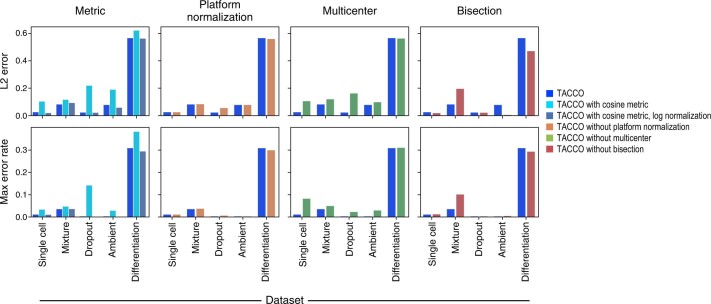
Effect of choice of cost matrix metric in OT and several booster options (platform normalization, multicenter, bisection) on annotation quality. L2 error (y axis, top; relevant for mixture annotations) and fraction of objects where the maximum annotation disagrees with the reference (y axis, bottom; relevant for classification problems) for different methods (colors) on different use cases (x axis). Baseline TACCO used the Bhattacharyya metric, platform normalization, multicenter, and bisection. Single cell: annotation of mouse colon scRNA-seq with itself as reference; Mixture: Gaussian mixtures of mouse colon scRNA-seq (Fig. 2a); Dropout: simulated Dropout dataset (Fig. 3a); Ambient: simulated ambient dataset (Fig. 3b), Differentiation: hematopoiesis scRNA-seq (Fig. 3c).

To address concrete biological perturbations and experimental variations between the new and reference data, TACCO provides a set of generic boosters (Extended Data Fig. 1), including (1) platform normalization (as in robust cell type decomposition (RCTD)^8^), which introduces scaling factors in the transformation between experimental platforms (for example, Drop-seq^25^ to Slide-seq^5^); (2) subclustering with multiple centers to capture within-class heterogeneity and (3) bisectioning for recursive annotation, assigning only part of the annotation in each step and working with the residual in the next step to increase sensitivity to subdominant annotation contributions (Methods).

TACCO is also equipped with different analysis tools that leverage the obtained compositional annotations (Fig. 1d). It adapts categorical annotation analyses to be applicable to mixture annotations across multiple samples, such as the quantification of spatial co-occurrence^26^ of annotations to analyze long- and short-range spatial structure, and calculates enrichments of annotations. For spatial data, TACCO can combine spatial and annotation information to define regions with similar annotation compositions across samples. Moreover, TACCO can split compositionally annotated expression data (for example, cell-type annotated Slide-seq beads) into categorically annotated expression data (for example, split beads per cell type) using a matrix scaling algorithm, yielding data that are amenable to standard downstream single-cell analysis workflows.

We evaluated TACCO on the following four different use cases: (1) decomposing cell-type fractions from spatially convoluted gene expression (in silico mixed scRNA-seq profiles for benchmarking and decomposing colon Slide-seq^6^ bead measurements); (2) inferring the source cell types of imaged single molecules (without requiring segmentation of cell boundaries from images) for the somatosensory cortex imaged with osmFISH^27^; (3) recovering cell type for scRNA-seq data with harsh dropout and with ambient RNA contamination (on simulated data) and (4) predicting differentiation fates of early hematopoietic progenitor cells^28^. As we show below, TACCO consistently achieved performance comparable to or better than other benchmarked methods and excelled especially in speed and memory consumption.

### Accurate decomposition of in silico mixed expression

**Fig. 2:**
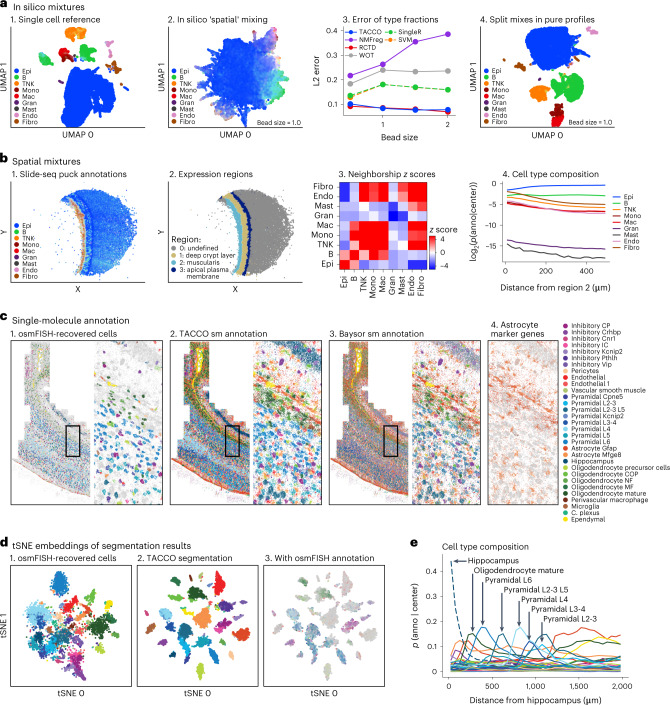
TACCO compositional annotation, cell segmentation and analysis of spatial expression data. **a**, Analysis of in silico mixtures. UMAP embedding of scRNA-seq profiles of mouse colon (1)^29^ and of in silico mixed scRNA-seq data before (2) and after (4) applying TACCOs splitting procedure into pure contributions. (3) L2 error (*y* axis) of cell-type annotations for each simulated bead size (*x* axis). Dashed lines: categorical annotation methods. **b**, A Slide-seq puck of normal mouse colon^29^ colored by TACCO cell-type annotations (1, colors are weighted and summed per bead) or by TACCO-defined regions (2) (Methods; Extended Data Fig. 3); (3) short-range (up to 20 µm) neighborship enrichment *z* scores over randomly permuted annotation assignments; (4) long-range (up to 500 µm) dependence of the composition of cell types around beads (log_2_(*p*(annotations|center)); *y* axis) at different distances from region 2 (muscularis; *x* axis) for each cell-type annotation (color). **c**, Comparison of TACCO and Baysor for single-molecule cell-type-of-origin annotation performance based on the single-molecule FISH: (1–3) Entire section profiled by osmFISH^27^ (left) and zoom-in on a small region (right) colored by (1) the published annotation of cells from watershed-based segmentation of the poly(A) signal or (2,3) the segmentation-free single-molecule annotation for cell-type-of-origin by TACCO (2) or Baysor (3) (Extended Data Fig. 6a); (4) measured astrocyte marker gene expression on the zoom-in as ground truth for the astrocyte annotation in (1–3). Gray molecules are unannotated. **d**, Effective cell segmentation of osmFISH data by TACCO. tSNE embeddings of RNA profiles in cell-like objects from the published watershed-based segmentation colored by the published cell-type annotation (1), from TACCOs annotation-based segmentation (using the annotations in c (2)) (2, 3), colored by either TACCOs single-molecule annotations summed per cell (2) or by the published segmented cell annotation pulled back to single molecules (as in c(1)) and summed per cell (3) (Methods; Extended Data Fig. 5). **e**, Recovery of layered tissue structure in osmFISH data. Long-range (up to 2,000 µm) dependence of the composition of cell types (*p*(annotations|center = hippocampus), *y* axis) recovered by TACCO for TACCO-segmented objects at different distances from the hippocampus region annotation for each cell-type annotation (Extended Data Fig. 6c).

**Extended Data Fig. 2:**
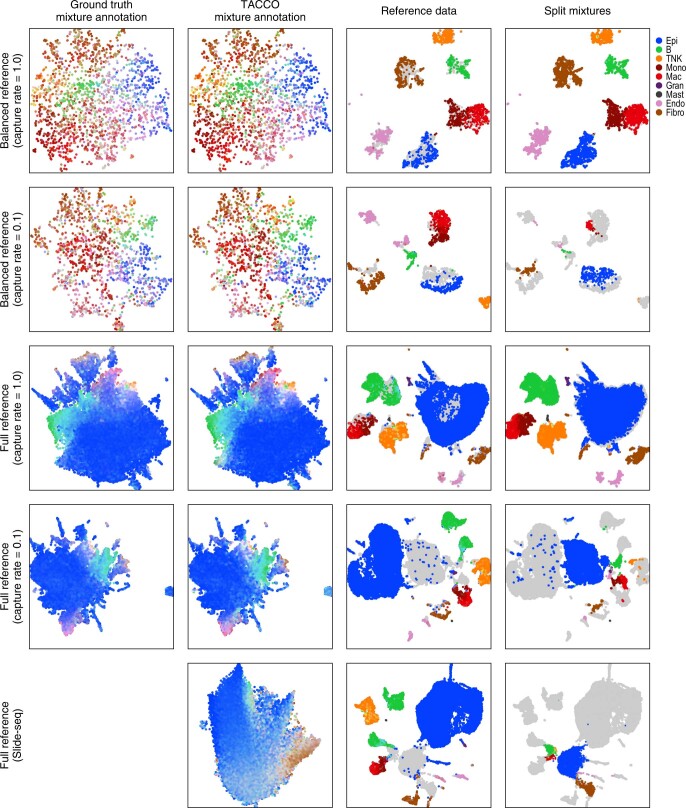
TACCO splitting of mixed expression profiles into pure constituents. UMAP embeddings of in-silico mixed mouse colon scRNA-seq profiles (top four rows: real or balanced type composition and with and without downscaling the count-yield per cell by a factor of 10) or Slide-seq beads (bottom row), based on ground truth (far left, where available), TACCO annotation (second left), reference data (second right) and mixed expression data split using TACCO’s annotation as input embedded in a joint UMAP of the concatenated reference-split dataset (rightmost). The in silico mix uses a beadsize of 1.0. For the joint embedding data was filtered to include only observations with at least 30 counts in genes which were annotated highly variable in the reference dataset, except for capture_rate = 1.0 where a threshold of 100 is used.

We first validated TACCO on simulated Slide-seq data that were generated from an annotated (real) scRNA-seq atlas of the mouse colon^29^ (Fig. 2a) as weighted mixtures of cells drawn from the reference atlas with Gaussian weights parameterized by bead size (Fig. 2a, Methods). We measured the L2 error between the ground-truth weights and the cell-type fractions inferred by TACCO for varying bead sizes. TACCO matched in reconstruction accuracy with RCTD^8^ and consistent with this outperformed all other tested state-of-the-art methods (Fig. 2a). Moreover, TACCO was much faster and had lower memory consumption than RCTD because RCTD fits a detailed model dense in parameters. For example, at bead size 1.0 (that is, bead and cells are of comparable size), the L2 errors were 0.081, 0.26 and 0.084 for TACCO, NMFreg^5^ and RCTD, while TACCO and RCTD runtimes were 84 s and 1469 s, and memory consumption was 2.7 GB and 10.4 GB, respectively. After annotation, TACCO uses the mean reference profiles and the compositional annotation to distribute the actual counts per gene and observation between the cell types and thereby generates separate observations per cell type, keeping the full gene space intact. A subsequent dimensionality reduction then recovers the low-dimensional structure of the reference data (Fig. 2a and Extended Data Fig. 2).

### Decomposition and structural analysis of Slide-seq data

**Extended Data Fig. 3:**
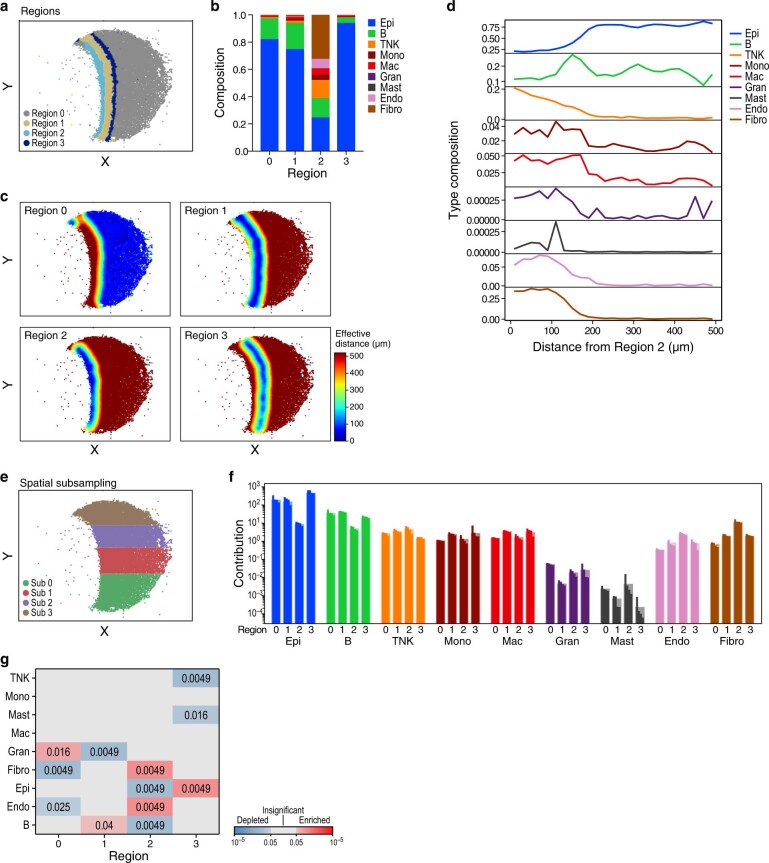
Example workflow and visualizations for spatial data analysis in TACCO for the mouse colon dataset from the ‘Spatial mixture’ task (Fig. 2b). (**a**) Slide-seq puck with beads colored by regions defined by joint expression space and position space clustering. (**b**) Fraction of cells (y axis) of each type (color) in each region (x axis). (**c**) Slide-seq puck colored by the effective distance of each bead from each region. (**d**) Density (y axis) of cell type annotations at different effective distance from region 2 (x axis). (**e**) Slide-seq puck colored by spatial split of the puck along a selected coordinate axis into multiple biological replicates for statistical enrichment analysis. (**f**) Geometric mean normalized cell type fractions (y axis) for each cell type (x axis) from each region (1–4) and subregion (per spatial split as in e; individual bars). (**g**) Enrichment p-values (colors) of the normalized cell fractions in (f) (Benjamini-Hochberg-corrected one-sided Mann-Whitney-U test across spatial splits).

We next applied TACCO to the annotation of real Slide-seq data from mouse colon with matching scRNA-seq^29^ (Fig. 2b and Extended Data Fig. 3). The different characteristics of scRNA-seq and Slide-seq data present annotation challenges. For example, cell composition can vary substantially, due to selection bias (much larger region assayed for scRNA-seq than Slide-seq) or because of differential capture efficiencies (for example, lower capture of fibroblasts by scRNA-seq^30^ compared to their true tissue prevalence). Moreover, Slide-seq’s low RNA capture rate yields much sparser data than scRNA-seq. Despite these challenges, TACCO recovered the expected layered structure of the colon along the muscularis–apical axis, short-range neighborship relations (closeness of stromal and immune cells with segregating epithelial cells) and long-range gradients on tissue structure scale from muscularis to apical plasma membrane (Fig. 2b and Extended Data Fig. 3).

**Extended Data Fig. 4:**
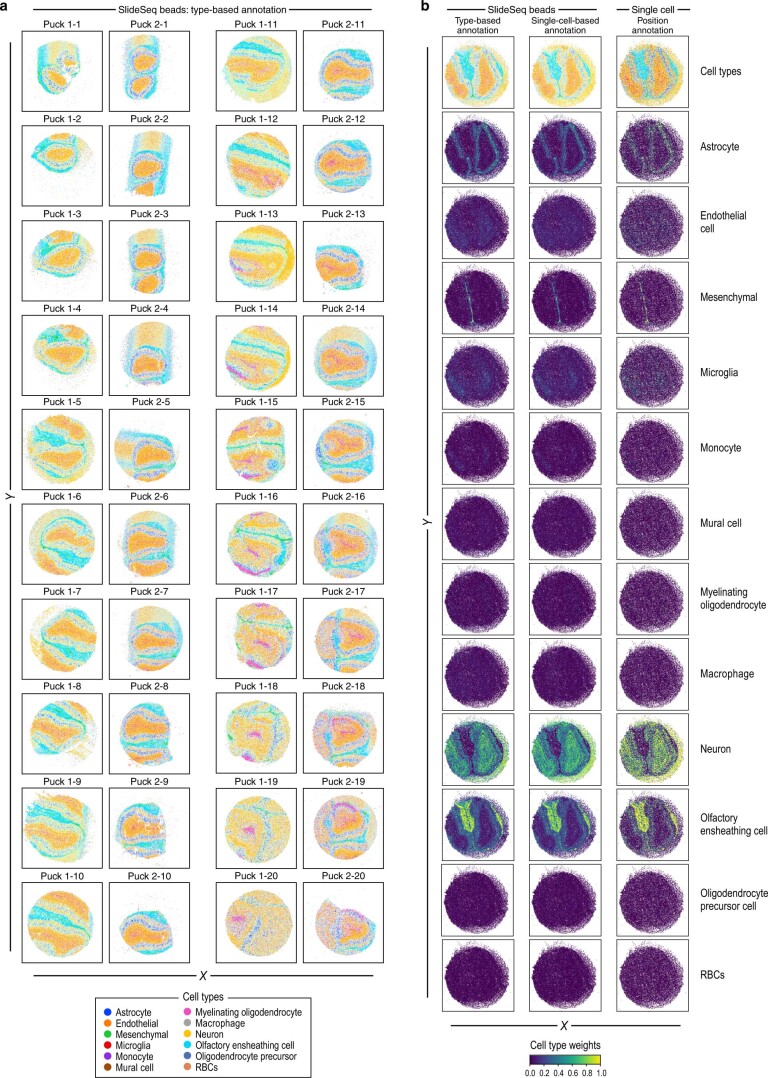
Annotating mouse olfactory bulb Slide-seq data^31^ using an scRNA-seq data^32^ with TACCO. (a) Compositional cell type annotation. Annotations (color legend) from a single TACCO run on 40 Slide-seq pucks across 2 replicates (replicate 1 in 1st and 3rd columns, replicate 2 in 2nd and 4th columns); (**b**) Comparison of different annotation strategies. Compositional cell type annotation (first row, color legend) and normalized cell type weights (remaining rows, color bar) from either direct compositional annotation of the spatial data (left column; default TACCO workflow, as in (a)); compositional single cell annotation of the spatial data, and deriving the cell type annotation from the single cell cell-type annotation (middle column); or positional annotation of the single cells in the reference with plotting the categorical cell type annotation at the positions of the beads with the maximum weight per cell (right column).

TACCO scales to real-world applications, such as annotating a large dataset of 40 Slide-seq pucks of the mouse olfactory bulb^31^. Using mean expression profiles of different cell types from scRNA-seq dataset from the same tissue^32^, TACCO annotated the full dataset of >1.4 million spatial observations on 8 CPU cores in under 12 min, yielding consistent results between consecutive pucks and two replicates (Extended Data Fig. 4a). As an alternative to cell-type mean profiles, TACCO can also use every individual cell profile as reference, similar to spatial reconstruction^12,13^ (Extended Data Fig. 4b) and infer the cells’ spatial arrangement, by either a compositional annotation of beads with all single cells or by annotating the single cells with the most likely position in the puck. Each of the three approaches implemented with TACCO (using mean expression profiles, compositional annotation with cells or mapping cells to most likely position) gave qualitatively similar spatial distributions of cell type; however, using mean profiles was more efficient computationally, while annotating cells with unique positions introduced additional noise in the spatial cell-type distribution.

### Single-molecule cell-type assignment and segmentation

**Extended Data Fig. 5:**
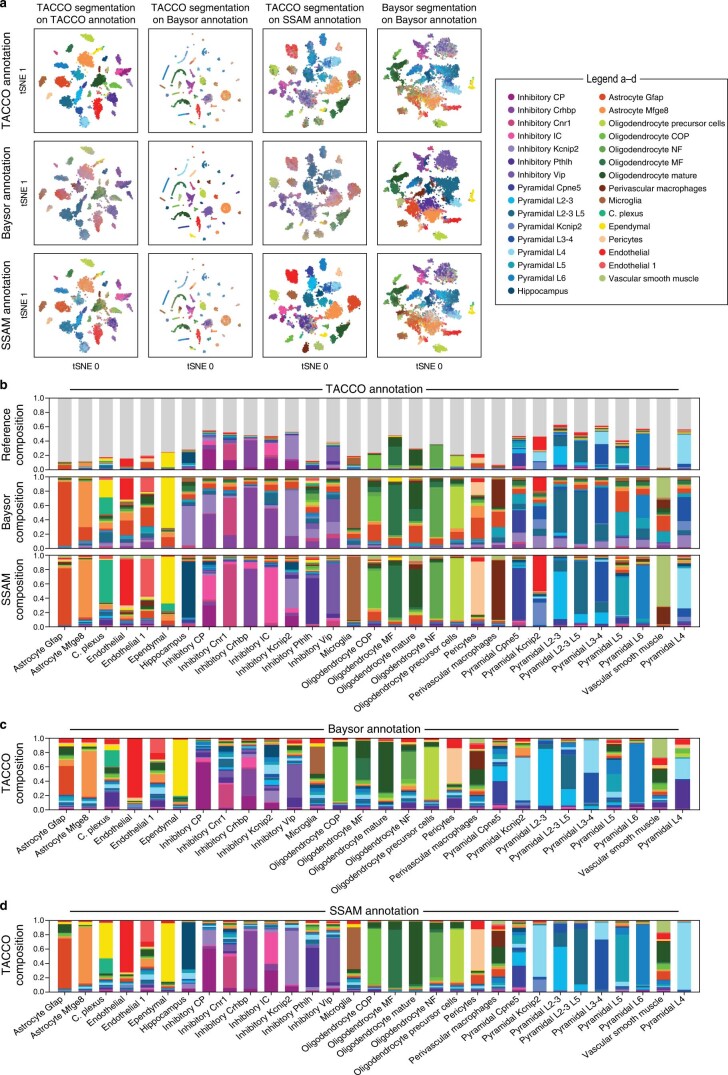
Comparisons of single-molecule annotations for osmFISH^27^. **(a)** tSNE of the expression profiles for objects (dots) resulting from different combinations of annotation (TACCO, Baysor^39^, SSAM^36^) and segmentation (TACCO, Baysor) methods (columns), colored by the aggregated single-molecule annotation from TACCO, Baysor, and SSAM (rows); **(b)** Distribution of annotations from the reference, Baysor and SSAM over the TACCO single molecule annotations; **(c,d)** Distributions of TACCO’s single-molecule annotations with respect to Baysor (c) and SSAM (d) single-molecule annotations.

**Extended Data Fig. 6:**
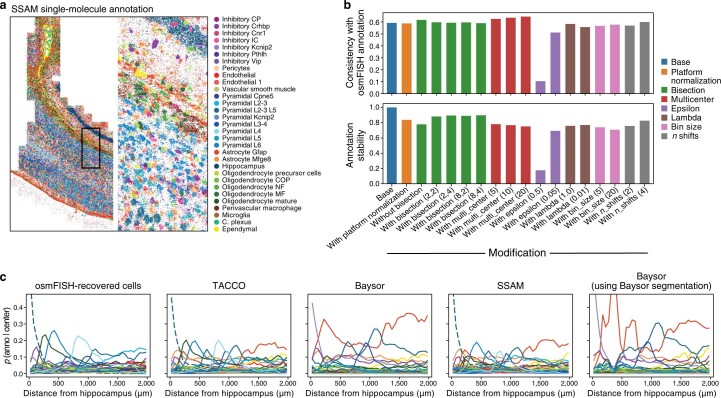
Analysis of single-molecule annotations for osmFISH^27^. **(a)** SSAM^36^ pixel-based annotation mapped to single molecules (presented like TACCO’s and Baysor’s^39^ single-molecule annotations in Fig. 2c (2,3)): Entire section profiled by osmFISH (left) and zoom-in on a small region (right) with the positions and mapped cell-type annotation of each measured molecule. **(b)** Impact of different choices (x axis) for different parameter categories (color) on TACCO’s single molecule annotation, based on consistency with annotation from osmFISH-recovered cells (y axis, top) or with the base configuration of the annotation, which is used in all other single molecule analyses with TACCO (y axis, bottom). **(c)** Comparison of cell type composition analysis (p(annotations|center = hippocampus), y axis) at different distances from the hippocampus region annotation (x axis) for each cell type annotation (color) from different methods (label top), all using a single cell type per segmented object; unlike in Fig. 2e), the hippocampus region annotation from osmFISH-recovered cells is used for all panels, as Baysor did not recover any hippocampus annotation.

Next, for single-molecule, high-resolution spatial imaging data (for example, MERFISH^33^ or osmFISH^27^), TACCO includes a wrapper for annotating individual, spatially imaged molecules with cell types, without prior cell segmentation. Standard procedures first segment the captured molecules either based on an image of the cells^27,33–35^ or by finding local maxima in spatially blurred expression fields^36^. Both can be challenging due to high cellular density, irregular cell shapes and misplaced molecules^37,38^. TACCO circumvents these limitations by annotating each molecule. To this end, TACCO bins molecules using a Cartesian grid into spatial neighborhoods, computes cell-type annotations for each neighborhood using the reference profiles (as for Slide-seq above) and assigns each molecule an annotation in a manner that recapitulates the neighborhood’s annotation fractions (Methods). Applied to a mouse brain osmFISH^27^ dataset, TACCO successfully accounted for each molecule (Fig. 2c), compared to only 36% of molecules annotated following cell segmentation and cell-type classification^27^ (Fig. 2c). Moreover, TACCO matched 59% of the molecular annotations in the original study, substantially better than Baysor^39^, a recent single-molecule annotation method based on Bayesian mixture models (Fig. 2c and Extended Data Fig. 5; Methods) (35% matched) and comparable to SSAM^36^, which annotates kernel density estimates of the expression on a Cartesian grid (Methods) (57% matched). Notably, TACCO was much faster than both methods (TACCO: 2 min; Baysor: 27 min and SSAM: 68 min; Extended Data Fig. 6a) with robust success over a wide range of parameter choices (Extended Data Fig. 6b). For cells with nonspheroid shapes, such as astrocytes, single-molecule annotation is especially advantageous; while TACCO, Baysor and SSAM annotate all molecules, the baseline segmentation and classification approach only accounts for 17% of astrocyte marker molecules (Fig. 2c).

Subsequent image-free segmentation to cell-like objects based on spatial and annotation information allowed TACCO to recover expression profiles with cell-type annotations, thus utilizing more of the available data and better representing distinct cell types than the baseline as indicated by a higher silhouette score for TACCO than the baseline (0.24 compared to 0.06; Methods; Fig. 2d and Extended Data Fig. 5). Applying TACCO’s segmentation on Baysor’s annotation yields an even higher silhouette score (0.45) but at the cost of missing three categories in the annotation (Hippocampus, Inhibitory IC and Inhibitory Pthlh) and a large fraction of molecules in very small segmented objects (28.7% of molecules in objects with less than 20 molecules, compared to 8.3% for TACCO) resulting from a less spatially homogeneous single-molecule annotation of Baysor compared to TACCO (Fig. 2c and Extended Data Fig. 5). Using Baysor’s built-in segmentation instead resulted in a silhouette score (0.03) that was worse than baseline and a longer runtime (50 min versus 3–5 min for TACCO segmentations). Replacing TACCO’s annotation with SSAM’s in the TACCO segmentation yielded a comparable silhouette score (0.22).

TACCO’s downstream analyses can use this image-free segmented single-molecule dataset to evaluate short- and long-range spatial patterns by computing the cell-type composition as a function of the distance to specific annotated cells. For example, we demonstrate this by recovering the layered structure of the mouse brain by calculating the distance to annotated hippocampal cells (Fig. 2e). Thus, TACCO’s spatial analyses efficiently bridge spatial scales from single-molecule data to tissue scale. TACCO’s downstream analysis is generic and can be applied to the annotations and segmentation derived from other methods as well. For example, TACCO- and SSAM-based analyses reproduce the layered structure, but the TACCO-based analysis yielded clearer cell-type density peaks than the SSAM-based one, while Baysor-based analyses were less conclusive (Extended Data Fig. 6c).

### Classifying cell types in the presence of technical artifacts

**Fig. 3:**
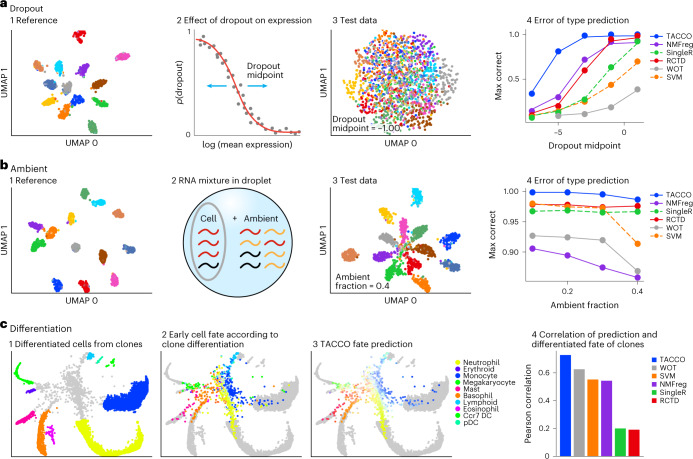
TACCO addresses dropouts, ambient RNA and continuous annotations. **a**, Dropout task. (1) UMAP embedding of the simulated scRNA-seq profiles without dropout; (2) schematic of the probability of dropout (*y* axis) for genes with different mean expressions (*x* axis); (3) UMAP embedding of the simulated data from (1) but with dropout; (4) fraction of correctly annotated cells (*y* axis, defined by the agreement of annotated type with maximal probability and the cell’s true annotation) at different dropout rate (*x* axis) for different methods (colors). Dashed lines: categorical annotation methods. **b**, Ambient RNA task: (1) UMAP embedding of simulated scRNA-seq profiles without ambient contribution; (2) schematic of test data generation, where ambient RNA is added to the cell’s expression profile; (3) UMAP embedding of simulated scRNA-seq profiles from (1) but with additional ambient contribution; (4) fraction of correctly annotated cells (*y* axis, defined by agreement of annotated type with maximal probability and the cell’s true annotation) at different levels of ambient RNA contamination (*x* axis) for different methods (colors). Dashed lines: categorical annotation methods. **c**, Differentiation task: (1–3) Spring plot of scRNA-seq profiles from hematopoiesis^28^ colored by cell types at day 4 and 6 (1); eventual clonal fate of day 2 cells (2), TACCO-predicted clonal fate of day 2 cells (3); (4) Pearson correlation coefficient (*y* axis) of predicted and actual fate (as evaluated in ref. ^28^) for each method (colors).

While TACCO is primarily a compositional annotation algorithm, it can be used as a cell-type classifier by interpreting the compositional annotation as probabilities and quoting the class with maximum probability as the classification result. Indeed, TACCO performed well in recovering the ground-truth cell-type classification of simulated data with either high dropout rates or high levels of ambient RNAs, as compared to bona fide classifiers, SingleR^17^ and SVM, and other compositional annotation methods turned into classifiers (Fig. 3a). Specifically, for data with high dropout rates, we simulated a reference dataset of distinct cell types using scsim^40,41^, where the probability of dropout is a function of log mean expression^42^. Although each cell’s type is preserved, increasing this technical noise leads to increasingly ‘fuzzy’ cell-type-specific signals. Based on the fraction of correctly labeled cells, TACCO outperformed both the classification methods and other compositional methods used as classifiers, with larger performance margins in TACCO’s favor for higher dropout rates. In particular, SVM, a top-performing cell-type classification method^43^, performed poorly when the test data shifted in gene expression. For example, on the same in silico dataset with mild dropout rate (dropmidpoint = −1), TACCO correctly assigned 99.9% of cells, whereas SVM only captured 44.7% correctly. For ambient RNA contributions (Fig. 3b), we simulated a reference dataset of distinct cell types using scsim^40^ with the ambient RNA model from CellBender^44^. TACCO outperformed all other baseline methods, based on the fraction of correctly labeled cells (Fig. 3b).

### TACCO-based prediction of cell fate decisions from scRNA-seq

TACCO also resolved continuous biological variability in the context of cell differentiation. To this end, we used a recent dataset^28^ based on the LARRY method, where hematopoietic stem and progenitor cells were clonally barcoded and clones were followed through subsequent cell division and differentiation, for cells profiled by scRNA-seq at day 2, 4 and 6. Most early cells (day 2) (96%) were undifferentiated^28^, while day 4 and 6 cells were mostly (61%) differentiated with distinct profiles; thus, for early progenitor cells, we construct a proxy of their fate identities based on the distribution of the annotations of their clonal relatives (linked via a shared barcode; Fig. 3c). We then challenged TACCO to predict the fates of early day 2 cells (test data) from the cell-type labeled expression of later, more differentiated cells (reference data). TACCO’s predictions are most correlated with the proxy clonal fates (Pearson’s *r* = 0.73), outperforming five other available methods (*r* = 0.19–0.62; Fig. 3c).

**Extended Data Fig. 7:**
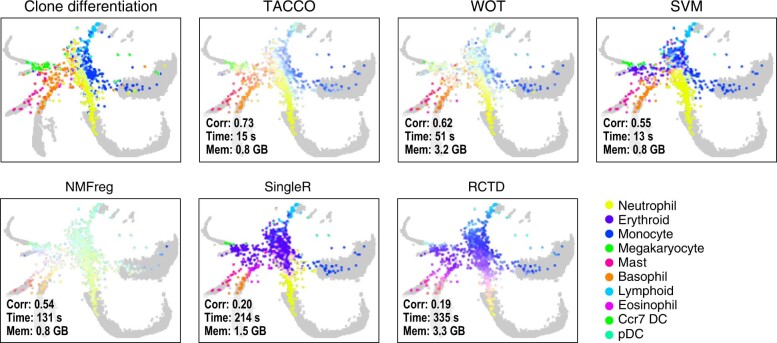
Comparison of methods performance on the differentiation task. scRNA-seq profiles (dots) from a hematopoiesis dataset^28^ colored by the eventual clonal fate of day 2 cells (leftmost) and by the predicted clonal fates of day 2 cells, for each method from Fig. 3c (4)). Reference profiles from differentiated cells (day 4 and 6) are in gray.

This difference in prediction accuracy impacted the biological features visible from the analysis. TACCO highlighted the uncertainty of early fate decisions and the correct commitment of cells from late stages, along with differentiation ‘watersheds’ at appropriate points, such as between basophils and neutrophils and between neutrophils and monocytes. Conversely, other methods either did not reproduce the watersheds (for example, WOT), did not reflect fate uncertainty at early stages (for example, SingleR), or did not capture the commitment of later stages (for example, NMFreg) (Extended Data Fig. 7).

### Benchmark of accuracy, runtime and memory requirements

**Fig. 4:**
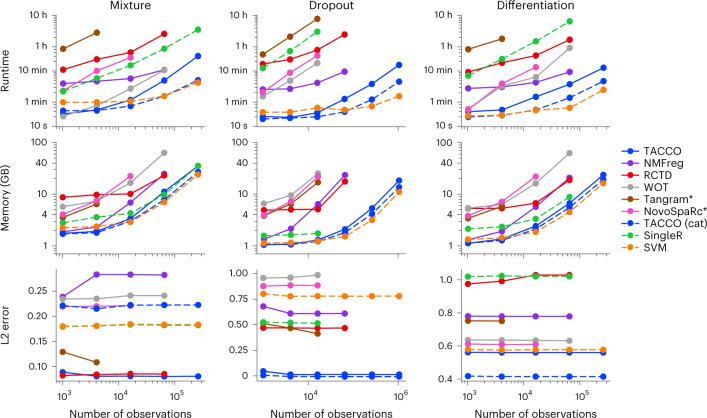
Benchmarking TACCO’s runtime, memory requirement and accuracy of annotation transfer. Runtime (top), memory (middle) and L2 error (bottom) of each method (colors) in the ‘mixture’ (left; with bead size = 1.0), ‘dropout’ (middle; with drop midpoint = −1.0) and ‘differentiation’ (right) use cases, at different numbers of observations (cells or beads, *x* axis), ranging from 2^10^ ~ 1 k to 2^20^ ~ 1 M, which are up/down sampled from the datasets in Fig. 2. Reference size is fixed at 2^14^ ~ 16 k. The maximum compute resources per run are 8 CPU cores for 8 h with 8 GB memory each. Missing data points indicate that either compute time or memory was insufficient to complete the annotation. Methods with an asterisk do not natively return (fractional) annotations of spatial measurements, which leaves the total annotation fractions in the spatial measurement as degrees of freedom. The wrapper fills that using the reference type fractions. Categorical annotation methods are dashed.

**Extended Data Fig. 8:**
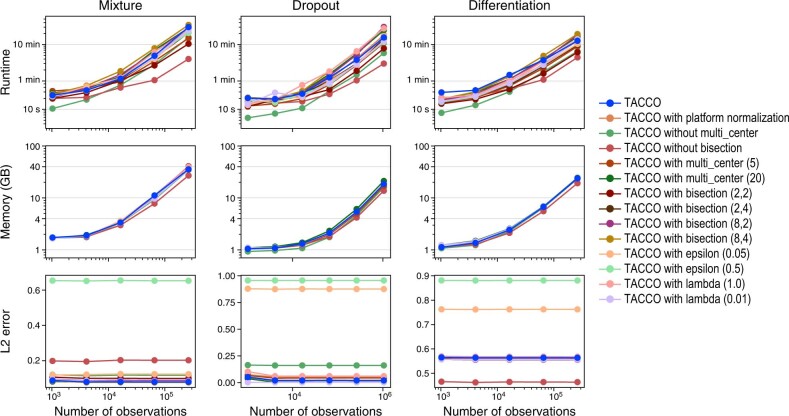
Impact of parameter variations on TACCO’s runtime, memory requirement and accuracy of annotation transfer. Runtime (y axis, top), memory requirement (y axis, middle) and L2 error (y axis, bottom) of each parameter choice (colors) in the ‘Mixture’ (left; with beadsize = 1.0), ‘Dropout’ (middle; with dropmidpoint = −1.00) and ‘Differentiation’ (right) use cases, at different numbers of observations (cells or beads, x axis), ranging from $2^{10} \approx 1k$ to $2^{20} \approx 1M$, which are up/down sampled from the datasets in Fig. 2. Reference size is fixed at $2^{14} \approx 16k$. The maximum compute resources per run are 8 CPU cores for 8 hours with 8 GB memory each.

**Extended Data Fig. 9:**
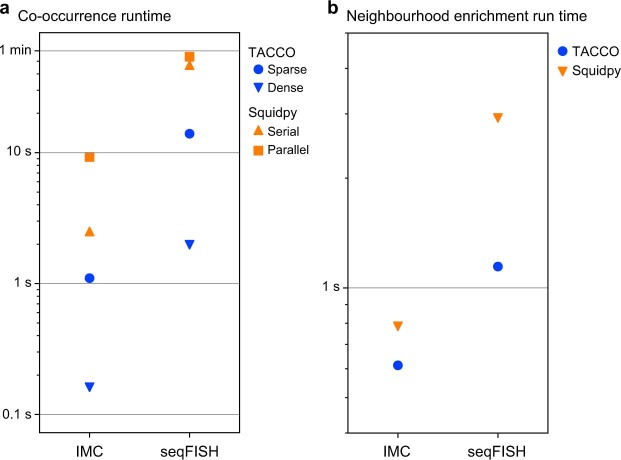
Runtime comparison of TACCO and Squidpy^26^. Run time (y axis) co-occurrence (a) and neighborhood enrichment (b) analyses for TACCO (blue) and Squidpy (orange). Datasets ‘imc’ and ‘seqfish’ are example datasets provided with Squidpy.

We designed TACCO with a focus on practical usability. In addition to its broad applicability to many use cases, TACCO has a modest resource footprint in terms of computing time, memory requirements and specialized hardware needs. We benchmarked TACCO’s computational requirements relative to those of baseline methods on standard x86 hardware on the ‘dropout’, ‘mixture’ and ‘differentiation’ tasks with a range of datasets sizes (10^3^–10^6^ observations; Fig. 4). Among all compositional annotation methods in the comparison, TACCO has the lowest runtime and memory requirements, often outperforming the other methods by an order of magnitude or more. Only plain SVM, a categorical annotation method, achieved comparable or better runtime and memory requirements depending on the problem size. However, for categorical annotation, TACCO can also run without the bisectioning booster which improves TACCO’s runtime even further. TACCO achieves all that while maintaining a stable and very competitive L2 error of the annotated compositions with respect to the ground truth across tasks and dataset sizes. While TACCO is very flexible and can be tuned to perform particularly well for specific use cases, a single configuration generally gives a decent performance in terms of runtime, memory and accuracy across various use cases (Extended Data Fig. 8; Methods). Additionally, runtime and memory usage are also optimized in TACCO’s downstream analysis tools. For example, similar statistics of co-occurrence and neighborhood-enrichment testing are computed via Squidpy^26^ and TACCO, but faster to compute with TACCO (Extended Data Fig. 9; Methods).

## Discussion

In conclusion, TACCO is a compositional annotation approach and broader analysis package rooted in the realization that multiple tasks in high-dimensional biological representations rely on successfully resolving continuous mixtures over cells or molecules, either due to biological or technical reasons. To leverage annotations of a reference dataset to annotate another dataset, TACCO integrates core, interpretable annotation methods, such as OT, and computational manipulations addressing concrete data perturbations. Together, these yield a top-performing framework in terms of accuracy, scalability, speed and memory requirements, making TACCO applicable for a wide range of annotation tasks.

However, the flexibility and efficiency of TACCO’s OT-based annotation come at a price. It cannot incorporate diverse detailed knowledge about the data-generating process as methods from the (deep) variational inference class do. While certain applications may benefit from creating a specialized annotation method instead of TACCO, this often requires substantial manual effort, detailed knowledge of the system and computing resources.

A further limitation is the requirement of overlapping feature spaces for reference and target data. Therefore, to use TACCO’s annotation for the transfer of annotations between modalities (for example, RNA–protein, DNA accessibility/methylation–RNA), a translation between the modalities needs to be performed externally to TACCO.

TACCO could be further extended by modeling continuous reference annotations (for example, interpolating/extrapolating along a linear reference), by integrating additional priors such as spatial smoothness for annotating spatial transcriptomic data and by integrating manifold learning techniques to better approximate expression distances from reference profiles.

The analytical form of interpretable methods like OT also opens the door to theoretical work on the limitations and guarantees of projecting annotations from one data to another, leading to more informed method selection.

TACCO’s computational efficiency places it as a potential building block for the development of new ‘meta’ analysis tools which perform different tasks. A prominent example for this is already implemented within TACCO: TACCO’s single-molecule annotation relies on repeatedly performing compositional annotations, which is feasible due to its underlying efficiency.

While showing here several examples of compositional and categorical annotations, we anticipate that TACCO will help in deciphering many other datasets, such as in decomposing expression of overloaded scRNA-seq experiments (for example, MIRACL-seq^45^, which overloads single nuclei to capture more rare cells) and in deciphering cells with complex continuous states, such as T cell spectra.

## Datasets

### Mouse colon scRNA-seq and in silico spatial mixing

Mouse colon scRNA-seq data^29^, as available from https://singlecell.broadinstitute.org/single_cell/study/SCP2038 in raw counts format after basic quality filtering, with the provided cell-type annotations, was used to simulate spatial mixing by sampling uniformly spatial coordinates for cells and for simulated beads on a square. To make boundary effects smaller, periodic boundary conditions were employed. On the resulting torus, the minimal Euclidean distance was taken between cells and beads. To factor in the ‘shape’ of the beads, that distance was plugged into a kernel to compute the weights of contributions of each cell to a bead. From these weights, expression counts of cells and cell-type annotation of cells, expression counts of beads, cell-type fractions and count fractions belonging to each type were computed.

A Gaussian kernel was used to determine the weight of cell *c* in bead *b* as follows:

$$\left( {w_{{\mathrm{gauss}}}} \right)_{cb} = r\,{\mathrm{exp}}\left( { - 1/2\left( {d_{cb}/l} \right)^2} \right)$$

where *d*_*cb*_ is the Euclidean distance between the centers of cell *c* and bead *b*, *l* = 1/2 bead_size √*1/n*, and *n* cells. To capture the higher sparsity of the spatial data, the weight was scaled by a capture rate parameter *r* such that a cell contributes a fraction of *r* of its counts to a bead with distance *0*. *r* = 1.0 was used everywhere except where explicitly stated.

### Mouse colon scRNA-seq and Slide-seq data

scRNA-seq (as in the section above) and Slide-seq data of normal mouse colon were obtained from ref. ^29^. Raw counts reported for Puck 2020-09-14_Puck_200701_21 were used as available from https://singlecell.broadinstitute.org/single_cell/study/SCP2038.

### Mouse olfactory bulb scRNA-seq and Slide-seq data

scRNA-seq of mouse olfactory bulb was obtained from https://www.ncbi.nlm.nih.gov/geo/query/acc.cgi?acc=GSE121891 (ref. ^32^), and Slide-seq data of the same tissue from https://www.ncbi.nlm.nih.gov/geo/query/acc.cgi?acc=GSE169012 (ref. ^31^). Raw counts were used for both datasets.

### Single-molecule osmFISH of the mouse cortex

osmFISH data were obtained from http://linnarssonlab.org/osmFISH/availability/ ref. ^27^. Reference expression profiles and their corresponding annotations were obtained from osmFISH-segmented cell profiles provided in ‘osmFISH_SScortex_mouse_all_cells.loom’.

Raw mRNA locations were obtained from ‘mRNA_coords_raw_counting.hdf5’.

Preprocessing was performed using the procedure in https://github.com/HiDiHlabs/ssam_example/blob/master/osmFISH_SSp.ipynb. This includes (1) shifting and rescaling cell/RNA coordinates to match each other (with an RNA coordinate system); (2) removing RNA molecules of bad quality; (3) correcting incorrect gene names and (4) removing molecules outside the intended imaged frame.

‘polyT_seg.pkl’, which contains a mapping of osmFISH’s segmented cell to molecule, was used to project osmFISH’s cell annotation onto individual molecules (approximating the ground truth). To visualize annotations at single-molecule level (Fig. 2c), a window of x range (1,150, 1,400) and y range (1,000, 1,600) was used.

### scRNA-seq simulations with scsim with enhanced dropout

Scsim^41^ with its corresponding default parameters (set in notebook ‘Step1_Simulate.ipynb’, with changing deloc to 5.0) was used to simulate scRNA-seq data. The dropout step described in Splatter^42^ was implemented in Python, by fitting a sigmoid curve through genes’ log mean count and their cell fraction with zero reads, where the sigmoid is characterized by shape and midpoint parameters. To enhance dropout, the sigmoid was shifted (decrease its midpoint) and the adjusted dropout probability was computed. This probability was then used to binomially sample the observed counts.

### scRNA-seq simulations with scsim with ambient RNA

To simulate expression profiles with ambient RNA, scsim’s^40^ simulation of mean expression per cells and genes (termed ‘updatedmean’ in scsim and denoted in Splatter^42^ as *λ*, a matrix of the means for each gene and each cell) was combined with CellBender’s^44^ model of ambient RNA contamination and downstream sampling of counts.

Specifically, the mean expression of gene *g* and cell *n* (or here, the cell in drop *n*) was obtained from scsim as *λ*_*ng*_. For CellBender’s probabilistic model, the probability of having a cell in the drop *y*_*n*_ is set to 1 (because empty drops are not considered), and *ρ*_*n*_ is set to 0 because 0 reads are exogenous to the drop as we account only for ambient RNA (pumped into the drop) and not for barcode swapping. Thus, the CellBender model simplifies to

$$C_{ng}\sim {{{\mathrm{NB}}}}\left( {d_n^{{\mathrm{cell}}}\chi _{ng} + d_n^{{\mathrm{drop}}}\chi ^a_g,{\Phi}} \right)$$

The two models were combined as follows:

$\lambda _{ng} = d_n^{{\mathrm{cell}}}\chi _{ng}$ is the mean expression (similar to CellBender, scsim also uses a log-normal distribution of cell size)

$\lambda' _g = {\mathrm{avg}}_n\lambda _{ng} = \chi _g^a$ is the mean true count of gene *g*, that is, the ambient contribution of the gene

Given the fraction of ambient RNA, *f* ^drop^, and the library size, $d_\mu ^{{\mathrm{cell}}}$, and scale, $d_\sigma ^{{\mathrm{cell}}}$, used for sampling the cell size, $d_n^{{\mathrm{cell}}},d_n^{{\mathrm{drop}}}$ ~ logNormal($d_\mu ^{{\mathrm{cell}}}$ + log(*f*^drop^), $d_\sigma ^{{\mathrm{cell}}}$) is sampled (that is, the same variance is employed as used for sampling the cell content and the mean is set to log(exp($d_\mu ^{{\mathrm{cell}}}$) *f* ^drop^).

*Φ* is sampled as defined in CellBender

Thus, we sample

$$C_{ng}\sim {{{\mathrm{NB}}}}( {\lambda _{ng} + d_n^{{\mathrm{drop}}}\lambda' _g,{{{\Phi}}}} )$$

For comparability, the reference is generated in the same way (using negative binomial sampling instead of scsim’s Poisson sampling) but without adding ambient RNA.

### scRNA-seq of clone-labeled hematopoiesis cells

Normalized counts (‘stateFate_inVitro_normed_counts.mtx’, together with the corresponding gene names and cell metadata) and cells’ clone matrix (‘stateFate_inVitro_clone_matrix.mtx’) were obtained from the GitHub repository https://github.com/AllonKleinLab/paper-data/blob/master/ (ref. ^28^). Cells were filtered to those belonging to clones with cells on day 2 and on days 4 or 6. The fate bias for each cell on day 2 was computed from the fates of its clone on days 4 and 6 (normalized to 1). Differentiated cells captured on days 4 and 6 were then used as a reference for annotating cells of differentiated fate from day 2.

### TACCO configuration

With the exception of the analyses mentioned below, TACCO was generally applied with an identical parameter set across all analyses in this manuscript: core annotation method OT (default TACCO setting), basic platform normalization (default TACCO setting for OT), entropy regularization parameter epsilon 0.005 (default TACCO setting for OT), marginal relaxation parameter lambda of 0.1 (default TACCO setting for OT), four iterations of boosting with a divisor of three (default TACCO setting for OT) and multi_center = 10 (10 representing means for each type to account for variability with an annotation category). Aside from parameter stability analyses, the exceptions are as follows: (1) for Fig. 4, in addition to running TACCO with the default parameter values as described above, TACCO was also applied without boosting, using only the maximum annotation per observation as categorical annotation, and all other parameters identical as an optimized categorical annotation method ‘TACCO (cat)’. (2) For Extended Data Fig. 4, TACCO was used with TACCO’s default settings only, that is, all parameters identical to above but not using the multi_center booster. (3) For the single-molecule annotation of the osmFISH data, default parameters were used again, but no platform normalization was used because the reference shares identical platform effects with the data to annotate. The additional parameters specific to single-molecule annotation were set to bin_size = 10, reflecting the expected size of features, that is cells, of about 10 µm, and n_shifts = 3, as a tradeoff between speed and reduction of artifacts.

### Baysor configuration

Running Baysor on the osmFISH example with the osmFISH-specific configurations available in the github repository (https://github.com/kharchenkolab/Baysor) and adapting the Baysor code (https://github.com/kharchenkolab/Baysor/blob/master/src/cli/main.jl) to accept cell-type means achieved only 25% match with osmFISH’s annotations. Therefore, we adapted code and used parameters from the segmentation-free annotation transfer described in the BaysorAnalysis repository (https://github.com/kharchenkolab/BaysorAnalysis/blob/master/notebooks/segmentation_free/segmentation_free_allen_smfish.md) and increased Baysor performance to achieve 35% match with osmFISH original annotations. These parameters are mainly default parameters: we used the functions build_molecule_graph() with filter = false, append_confidence() with nn_id = 16, and cluster_molecules_on_mrf() with do_maximize = false, max_iters = 1,000, and n_iters_without_update = 20.

For the segmentation we followed the Baysor code (https://github.com/kharchenkolab/Baysor/blob/master/src/cli/main.jl) and used the specialized values for the osmFISH data from the repository, where available, and default values from the repository otherwise. Specifically, we used scale-std = ‘25%’ and prior-segmentation-confidence = 0.2 from https://github.com/kharchenkolab/Baysor/blob/master/src/cli/main.jl, iters = 500 and num-cells-init = 30,000 from https://github.com/kharchenkolab/Baysor/blob/master/examples/osm-FISH/README.md, scale = 70.0 and min-molecules-per-cell = 30 from https://github.com/kharchenkolab/Baysor/blob/master/configs/osm_fish.toml and new-component-fraction = 0.3 and new-component-weight = 0.2 from https://github.com/kharchenkolab/Baysor/blob/master/src/cli/common.jl.

### SSAM configuration

For SSAM, we followed the SSAM user guide and used default parameters except for find_local_max() for which we supplied the parameters recommended in the user guide: search_size = 3, min_norm = 0.027, min_expression = 0.2.

To map the SSAM annotations of the generated Cartesian grid back to individual molecules and to compare them with the single-molecule annotations from TACCO and Baysor, we assigned to every molecule the annotation of the grid point closest to it.

### RCTD configuration

For RCTD, we adapted the default parameters to work in the framework of TACCO such that all clusters in the reference are used (CELL_MIN_INSTANCE = 0), all cells end up with an annotation (UMI_min = 0), RCTD does not throw an exception for lowly covered types in the reference (UMI_min_sigma = min(300,median_observations_(total_counts_per_observation)-1)), and every observation gets a compositional annotation over all available types (doublet_mode = ‘full’).

### NovoSpaRc configuration

For NovoSpaRc, we used alpha = 1.0 and adapted the default parameters to use all available genes.

### Configuration of other annotation methods

For all other annotation methods (SVM, SingleR, NMFreg, WOT, Tangram), we used default parameters.

## Methods

### Overview of TACCO framework

TACCO is based on three guiding principles: modularity, interpretability and efficiency. TACCOs compositional annotation algorithm is built from a single fast core method, which is then supplemented by a diverse set of wrappers and ‘boosters’, each providing additional functionality and features. The framework is completed by a set of downstream analysis tools, some of which are especially optimized for analysis involving compositional annotations. TACCO relies on Anndata and seamlessly integrates with the Scanpy^46^ ecosystem.

### Compositional annotation

TACCO aims to annotate single cells and cell-like objects, like Slide-seq beads or Visium spots, with compositional annotations. In cases with discrete ground truth (for example, a B cell versus a fibroblast), we interpret the compositional annotation as probabilities of belonging to a category. Both objects *b* and categories *t* are in a common high-dimensional data space, for example, expression space. Generally, objects that are close to a category in that space should have a high contribution of that category in the compositional annotation.

The goal of the annotation is to find a matrix *ρ*_*tb*_ which gives the distribution of annotation over objects *b* and categories *t*. Some applications imply certain natural choices for the ‘units’ of *ρ*_*tb*_. For example, in the case of count data, the natural units are counts as well, such that *ρ*_*tb*_ gives the counts in object *b* which are attributed to category *t*. The marginal distribution over categories is just the total number of counts Σ_*t*_ *ρ*_*tb*_ = *ρ*_*b*_ per object *b* and is known. The marginal distribution over objects Σ_*b*_ *ρ*_*tb*_ = *ρ*_*t*_ is not known beforehand and is an output of the annotation process. As the marginals per object are known in general, it is equivalent to cite only the normalized distributions *ρ*′_*tb*_ = *ρ*_*tb*_/*ρ*_*b*_ with Σ_*t*_ *ρ*′_*tb*_ = 1 as an annotation result.

### Core annotation method by OT

TACCO’s core method is entropically regularized, partially balanced OT (an intrinsically fast variant of OT). Balanced OT solves the optimization problem *γ*_tb_ = argmin_*γtb*_ Σ_*tb*_ *γ*_*tb*_ *M*_*tb*_ under the positivity constraint *γ*_*tb*_ ≥ *0* and the marginal constraints Σ_*t*_ *γ*_*tb*_ = *c*_*b*_ and Σ_*b*_ *γ*_*tb*_ = *c*_*t*_ with the transport cost Σ_*tb*_ *γ*_*tb*_ *M*_*tb*_ = *<* *γ*, *M* > _F_ given by the Frobenius inner product of a mapping matrix *γ*_*tb*_ and a constant matrix *M*_*tb*_. *M*_*tb*_ encodes the cost of ‘transporting’ or mapping an object *b* to an annotation *t* and must be chosen sensibly to yield a ‘good’ mapping *γ*_*tb*_. The annotation problem is solved if the marginals *c*_*b*_ and *c*_*t*_ and the cost *M*_*tb*_ can be tuned such that *γ*_*tb*_ = *ρ*_*tb*_.

The marginal over annotations is known from the data *c*_*b*_ = *ρ*_*b*_, while *c*_*t*_ = *ρ*_*t*_ is not. This can be used to encode prior knowledge about the data, for example, from a reference distribution. To support the general case when such a reference distribution is not available, partially balanced OT is used: instead of fixing the type marginal exactly, a Kullback–Leibler divergence penalty is imposed scaled with a parameter *λ*. This can be used to tune the amount of trust put in the prior distribution.

Choosing a well-performing cost function is more ambiguous. The cost function uses the information in data space and assigns a dissimilarity to each object-category combination. A straightforward choice is the cosine distance, or, for expression count data, the cosine distance on normalized and log1p-transformed data. In our benchmarks, the cosine distance on transformed data led to better results (Extended Data Fig. 1), but the transformation is rather specific for count data. Inspired by the overlap of states in quantum mechanics, a different measure, the Bhattacharyya coefficients, is generally used, with similar performance and without direct reference to counts. Bhattacharyya coefficients are a general measure of the overlap of probability distributions and are defined as BC(*p*,*q*) *=* Σ_*g*_√*p*_*g*_*q*_*g*_ for two probability distributions *p* and *q* in the data space. To allow the user to adapt the method according to the needs of their particular application, TACCO implements several other metrics, as well as making all scipy metrics available.

OT’s optimization problem is in general nonconvex and numerically expensive. However, using entropic regularization makes it strictly convex and efficiently solvable by using the Sinkhorn–Knopp matrix scaling algorithm^47^. For entropic regularization, the tunable entropy regularization term *εΩ*(*γ*_*tb*_) *=* *ε* Σ_*tb*_ *γ*_*tb*_ log(*γ*_*tb*_) is added to the objective function. This term favors mappings that do not map a given object to a single annotation.

### Alternative annotation methods

The core, OT-based annotation method can be swapped by a number of other built-in methods, including nonnegative least squares or support vector machine (SVM) or by wrapped external methods from both Python and R, including NMFreg^5^ or RCTD^8^. Custom core methods can also be added using a functional interface. The wrapped external and custom functions may already include ‘booster’ functionality (below). For example, RCTD already contains platform normalization. Many of the simple built-in methods are amenable to hand optimization, for example, by supporting data sparsity consistently, which makes them even faster. All the built-in methods are generally optimized for standard x86 hardware, but wrapped external methods may use GPU acceleration (for example, Tangram^12^).

### Overview of boosters

Boosters can improve the performance of the core method or provide support for ‘missing features’ for example, for platform normalization, for using subtype variability to enhance the type representation in single-cell data, for creating a deconvolution method from a categorical annotation method and the other way round (Extended Data Fig. 1). They can be combined flexibly to adapt to special requirements for specific applications. The modularity introduced by boosters makes it straightforward to ‘unbox’ TACCO and understand what each part does. While most boosters do have some overhead in runtime, in the end, they all do the heavy lifting by calling the fast core method one or several times and transforming its inputs and/or outputs.

### Platform normalization

Dramatic differences in datasets can arise solely from differences in the experimental technique that impact the profiled cellular compartments (for example, single cell versus single nucleus), cellular compositions (due to differential capture of cells of different types) or gene biases. Annotation of data from one platform using a reference of another platform is much more difficult without accounting for these platform-dependent biases^8^. This booster can be safely disabled if no platform effects are expected.

A platform normalization step mitigates platform-specific effects by rescaling data from one platform to make it comparable to data from a different platform, separately in each dimension *g* of the data space, for example, separately per gene. The necessary rescaling factors are estimated from data. A related but simpler approach compared to that given in ref. ^8^ is pursued, making less assumptions and therefore usable across a broader range of applications.

The representations of the categories *t* as sets of vectors in data space $\pi ^A_{gt}\,{{{\mathrm{and}}}}\,\pi ^B_{gt}$ on the two platforms *A* and *B* are linked to each other via the platform normalization factors $f^{AB}_g\,{{{\mathrm{as}}}}\,\pi ^A_{gt} = f^{AB}_g\pi ^B_{gt}$. If the category representations $\pi ^B_{gt}$ for platform *B* and the (pseudo-bulk) category marginals $\rho ^A_t$ for platform *A* are known, the data space marginals $\rho ^A_g$ can be written as ${\rho^A_{g}} = {\mathop {\sum}\nolimits_{{{\mathrm{t}}}} {\pi ^A_{gt}\rho ^A_t}} = {f^{AB}_g}{\mathop {\sum}\nolimits_{{{\mathrm{t}}}} {\pi ^B_{gt}\rho ^A_t}}$, and therefore $f^{AB}_g = \rho ^A_g/\mathop {\sum}\nolimits_{{{\mathrm{t}}}} {\pi ^B_{gt}\rho ^A_t}$. The category marginals $\rho ^A_t$ are themselves usually a result of the annotation procedure and used here as input to a preprocessing step for the procedure. However, an iterative scheme can also be used. Starting with the assumption that the annotation marginals are identical to the reference (which is reasonable for example, if matched spatial and single-cell data are available), most of the gene-wise platform effect can be already captured. Next, platform normalization is rerun after a first round of cell typing using improved type fractions, and the process is iterated until the normalization factors are stable. In practice, this procedure converges very rapidly with the initial step being by far the most relevant one.

After determining the gene-wise platform normalization factors $f^{AB}_g$, they are used to rescale the data space in either the reference or the test data or equivalently to work in the units of the test or the reference data. As the probabilities and the resulting annotation distribution are given in terms of these units, choosing test data units or reference data units can lead to quite different results. Which option should be preferred depends on the use case and downstream analyses. Here, test data units are chosen to retain integer count data for object splitting.

### Multicenter

In many cases, there are multiple observations per annotation category in the reference dataset, as is the case in single-cell data. To integrate the variability of these observations while maintaining speed, the observations per category are subclustered with *k*-means clustering to get multiple mean profiles per category. The annotation function is then called with this subannotation, and its result is summed over the subannotations to give an annotation in terms of the original categories. When the reference is already finely annotated, no improvement is expected from this booster, which could then lead to worse performance, because less data are available per subannotation to average noise and the reference gets overfitted.

### Bisectioning

Some annotation methods can be biased toward low-entropy annotations such that they may overweigh dominant categories in mixtures (the extreme case is a categorical classifier). Such methods can be adapted to compositional annotation by running the annotation method iteratively. In each iteration, the annotation is not used as the final result, but instead, a certain fraction of it is used to subtract a reconstructed approximation of the data from the original data. The residual is again subjected to the annotation method, while a fraction of the annotation result is added to the final annotation result. This procedure is very similar to gradient boosting. Bisectioning is useful when the objects are additive mixtures of the annotations in the data space. If the data consist (mainly) of objects which are best described by a single annotation, this booster can decrease performance, for example, by resolving the ambient contribution in single-cell data.

### Choosing parameters

While a single configuration of TACCO generally gives decent performance for a wide variety of use cases (Extended Data Fig. 8), it can be tuned to perform particularly well for certain use cases. Relevant parameters for the tuning include the following components.

#### Boosting/bisectioning

TACCO’s default depends on the core annotation method. As OT itself lacks support for additive compositional annotations, TACCO activates this booster for OT by default. Higher values for the number of bisections and the bisection divisor generally lead to more precise compositional annotations but at the cost of increased runtime, which scales roughly linearly with the sum iterations+divisor. A divisor smaller than 3 can lead to sizable artifacts, and so a divisor equal to or greater than 3 is recommended.

#### Multicenter

By default, TACCO does not use the multicenter booster (setting a number of representing means for each annotation category), as it is useful only in cases where the annotation categories have a large within-category variation. However, as many reference datasets come with coarse annotation categories, the multicenter booster is activated for most analyses in this study. This booster is not recommended if the categories already cover the full biologically significant heterogeneity in the data or if the dataset has only a single representing observation per category. A value of 5 or 10 is recommended in other cases (as this generally captures the heterogeneity in many datasets). Larger values are generally not recommended as they lead to an increase in runtime and have the potential for overfitting, by using irrelevant variability in the data for the annotation process.

#### Platform_iterations

TACCO’s default depends on the core annotation method. As OT itself does not have built-in platform normalization and most pairs of reference and target data have nontrivial platform effects, basic platform normalization is the default for OT. It is generally recommended not to disable platform normalization, except for specific cases, for example, if part of the dataset of a single batch was manually annotated and the annotation should be transferred to the rest of the data.

#### Max_annotation

TACCO’s default is a compositional annotation with nonzero values simultaneously possible for all annotation categories per observation. Max_annotation can be used to set the number of nonzero values per observation. For categorical annotation, one might want to restrict this to a single nonzero value per observation, corresponding to setting max_annotation to 1, while for data with very few contributing cells per observation (for example, Slide-seq or scRNA-Seq data with doublets) a suitable value could be 2 or 3.

#### Marginal relaxation parameter lambda (OT-specific)

TACCO’s default value is chosen as a compromise between using prior information from the reference pseudobulk annotation composition (for example, cell-type proportions) and annotating in a fully data-driven manner. While larger values (larger cost of having a pseudobulk annotation composition different from the reference) can stabilize the annotation in cases of low correspondence between reference and target data, smaller values can give more precise pseudobulk annotation compositions if the two datasets are highly corresponding and do not exhibit batch effects that are not accounted for by platform normalization.

#### Entropy regularization parameter epsilon (OT-specific)

TACCO’s default is chosen such that the regularization’s effect on the results is minimized: it biases the results toward high entropy configurations, that is, every observation (for example, spatial observation) is assigned a compositional annotation with large contributions from different categories (for example, cell types). This parameter must be nonzero, as the efficient regularized OT solver breaks down at zero epsilon (and numerically already at epsilon much smaller than 0.005). Except for specific settings (that is, prior information external to the dataset itself) in which it is beneficial to bias the result toward significant contributions of many annotation categories in the result, this parameter should only be changed in rare cases of numerical instability—which we did not observe with TACCO’s default.

### Object splitting

The annotation method generates an assignment of a composition of annotation categories for every observation. Generally, each observation is associated with more than one category with nonzero contribution. When the categories are cell types, this can be problematic for downstream applications that require the expression profiles of single cells as input, that is, pure profiles that can be attributed to a single cell. For example, as cell-type-related expression constitutes a strong signal, analysis of expression programs is easier across cells of shared type. Thus, it is desirable to derive several ‘virtual’ observations for every real observation, which correspond to the pure contribution of each annotation category.

A similar idea was proposed in ref. ^8^, where the expected contribution of each cell type to the expression of one Slide-seq bead is approximated. In that approach, cell-type fractions that were reconstructed for every bead are taken as input in a Bayesian analysis, along with type-specific expression profiles and the bead-count matrix to yield expected reads per gene, bead and cell type. The result, however, lacks consistency with the annotation and the measured count matrix in the sense that the marginal over genes is not recovered. Here, we follow a similar strategy, but we directly integrate marginals as constraints of a matrix equivalence scaling problem in a data-driven frequentist approach.

In this approach, data generation is modeled as drawing single molecules labeled with gene *g*, cell type *t* and observation/bead *b* from the sample, which constitutes the central object—the joint probability *p*(*gtb*) for a given molecule to be of gene *g*, cell type *t* and bead *b*. In the actual experiment, only *g* and *b* are measured, yielding *p*(*gb*) *=* Σ_t_ *p*(*gtb*), the ‘t-marginal’. From the reference data, the reference profiles *p*(*g*|*t*) *=* Σ_*b*_ *p*(*gtb*)/*p*(*t*) are available, and from the annotation process the annotation result *p*(*tb*) = Σ_*g*_ *p*(*gtb*), the ‘g-marginal’ is obtained. Further, *p*(*gtb*) is modeled with a Bayesian-inspired product ansatz: *p*(*gtb*) = *p*(*gt*) *n*(*gb*) *n*(*tb*), with free parameters *n*(*gb*) and *n*(*tb*). These are fixed by enforcing the *t*- and *g*-marginals. The ansatz can be interpreted as adjusting the cell-type profiles and pseudobulk annotation with object-wise scaling factors per data dimension *g* and annotation category *t* such that the measurement and the annotations are reproduced exactly. To determine the parameters from the marginals, we have to solve a separate matrix equivalence scaling problem per object *b*.

Matrix equivalence scaling problems are guaranteed to have a solution if the matrix to be scaled, that is *p*(*gt*), has only positive entries. Therefore, a small positive number is added to all the elements of *p*(gt) to make the problem well defined. These problems can be solved by iterative normalization of the columns and rows of *p*(*gt*). This simple algorithm is known under many names, for example, the RAS algorithm^48^ or for doubly stochastic matrices as the Sinkhorn–Knopp algorithm^47^, and it is also the algorithm used to solve OT efficiently^49^. In contrast to OT, where there is a single matrix scaling problem, here a separate problem is solved for every object. Although this initially seems like a practical performance problem, these problems can be solved in parallel and use the same data, leading to speedups by reducing memory accesses. Moreover, we use the sparsity of the count frequency matrix *p*(bg) to implement the RAS algorithm very efficiently for the problem at hand.

By rescaling the resulting *p*(*gtb*) with the total weight per object (for example, total counts per cell for sequencing data), a consistent annotation-resolved split of the measurement is obtained, consisting of floating-point numbers. For expression count data, this split generally includes many values much smaller than 1. To optimize sparsity and obtain integer counts, an option is available to round this result by flooring and redistributing the remaining reads (via multinomial sampling from the remainders). The resulting split count matrix retains biological signal and can be used in standard downstream analyses (Extended Data Fig. 2).

### Single-molecule annotation

To annotate single molecules by cell-type assignment, TACCO first bins the single-molecule data in space to generate aggregate cell-like objects. TACCO then annotates them either using internal or wrapped external methods. Subsequently, TACCO maps the resulting compositional annotation back to the single molecules, as follows. First, object splitting is used to distribute molecules to annotations, resulting in a probability for every molecule species in the bin to have a certain annotation. This annotation is then distributed randomly among the molecules of that molecular species such that each molecule has only a single annotation and the population of molecules reproduce the annotation probability. Because spatial binning can introduce arbitrary boundary artifacts in the definition of local neighborhoods for molecules, TACCO repeats the binning and annotation for *N* (usually 2–3) spatial shifts of the Cartesian grid in steps of 1/*N* of the grid spacing per spatial dimension. For *d* spatial dimensions, this results in *N*^*d*^ annotations per molecule. The final annotation of each molecule is then determined by a majority vote. This simple single-molecule annotation is only feasible with fast annotation methods, which can be run multiple times on differently binned data in a reasonable amount of time.

In addition to the parameters for compositional annotation, the single-molecule annotation introduces extra parameters. We evaluated the annotation’s sensitivity to parameter value changes with respect to the baseline single-molecule TACCO configuration (Extended Data Fig. 6b). Sizable changes in most parameters conserved about 80% of the single-molecule annotations relative to the baseline and were consistent with the reference annotation of the osmFISH-recovered cells at about 0.6, with one notable exception as follows: increasing OT’s entropic regularization parameter epsilon leads to significant reductions in accuracy and stability as it drives the annotation to be homogeneous regardless of the data. As for compositional annotation, we therefore recommend keeping the small default value which is just large enough to make OT numerically stable in practice.

### Image-free segmentation

An image-free, density-based cell segmentation approach uses the per-molecule annotation to determine molecule assignments at critical cross-category boundaries. We assume that the exact assignment of boundaries within a category is not very important as long as it gives rise to a reasonable size of the segmented objects. The segmentation is implemented as graph-based hierarchical spectral clustering.

As the number of single molecules can easily be of the order of 10^9^, an efficient Euclidean sparse distance matrix computation is required. As scipy’s sparse distance matrix calculation is serial and too slow for this application, a custom, fast, parallel and still generally applicable algorithm was developed and implemented. After calculating the distances $d_{ij}^{{\mathrm{spatial}}}$ between molecules *i* and *j* in position space, a distance contribution is added in quadrature which is derived from the single-molecule annotation $d_{ij}^{{\mathrm{annotation}}}$ to get a combined distance: $d_{ij}^{{\mathrm{total}}} = \surd \left( {d_{ij}^{{\mathrm{spatial}}}} \right)^2 + \left( {d_{ij}^{{\mathrm{annotation}}}} \right)^2$. The annotation contribution can be either zero within an annotation and infinity between annotations, automatically derived using distances between the expression profiles of the annotations or manually specified. As the clustering works on affinities and not on distances, the distances have to be transformed into affinities, which is done by a Gaussian kernel with a subcellular distance scale.

As the number of molecules is so large, the clustering algorithm has to be fast and scalable, work entirely on sparse matrices and cut the neighborship graph at sensible places to generate reasonable cells-like objects. For this, a hierarchical clustering scheme was developed based on the spectral clustering implementation of scikit-learn as follows: it can handle sparse matrices, can use algebraic multigrid solvers for speed and, for only two clusters, solve the normalized graph cut problem^50^. The best cuts are found iteratively in top-down fashion (binary splitting the data at each iteration). To keep the dataset tractable for the initial cuts, the spatial structure of the data is used to generate supernodes in the graph before clustering. When the clustering comes down into the size regime of a few single cells, several heuristics are employed to determine whether to accept a proposed cut based on the shape and size of the clusters, and on comparing the affinity loss of the proposed cut to the expected cost for cutting a homogeneous bulk graph of corresponding size and dimension. The final result is another column of annotation for the molecules containing unique object ids.

For visualization, these objects are filtered to include at least 20 molecules, and the associated expression profiles are normalized as in ref. ^27^ and embedded by a tSNE. To evaluate the self-consistency of annotation and segmentation, the silhouette score of the cell-type annotations is evaluated on identically filtered and normalized profiles using the silhouette_score function from scikit-learn.

### Region definition

For spatially convoluted expression data, such as in spatial transcriptomics methods including Slide-seq and Visium, clustering (of beads or spots) in expression space is less meaningful as individual cells are mixed. ‘Regions’ which are defined both in position and expression or annotation space can be a meaningful alternative. TACCO implements a method to define such regions (Extended Data Fig. 3a), consisting of the following two steps: (1) construction of one *k*-nearest neighbors graph based on expression (or annotation) distances and another *k*-nearest neighbors graph based on physical distances; and (2) combination of the two graphs as a weighted sum of the graphs’ adjacencies. Putting more weight on the position space adjacency gives contiguous regions in position space, while more weight on the expression adjacency leads to separated islands of similar expression being annotated with the same region and can connect regions across different spatial samples (for example, Slide-seq pucks). To account for the missing links from the position graph between samples, the cross-sample adjacency is scaled up by a balancing weight factor. The combined adjacency matrix is then subjected to standard Leiden clustering^51^ which assigns consistent region annotation across samples.

### Colocalization and neighborhood analysis

To score colocalization or co-occurrence of annotations^26^, TACCO calculates *p*(anno|center;*x*)/*p*(anno), the probability to find an annotation ‘anno’ at a distance *x* from a center annotation ‘center’ normalized by the probability to find ‘anno’ regardless of a ‘center’. This is well defined also for noncategorical annotations, which are commonplace for the compositional annotations created with TACCO and for pairs of unrelated annotations.

As the most time-consuming computations for co-occurrence are the same as for neighborhood-enrichment analyses, TACCO also calculates neighborhood-enrichment *z*-scores^52^ for a set of distance bins (as opposed to the set of direct neighbors on a graph) and again supports noncategorical annotation, pairs of unrelated annotations for ‘anno’ and ‘center’, and multiple samples while being competitive performance-wise (Extended Data Fig. 9).

### Annotation coordinates

To analyze a given annotation (for example, cell-type composition) with respect to its spatial distance from a reference annotation (for example, histological annotation; Extended Data Fig. 3b,c), TACCO implements an algorithm that determines a stable ‘annotation coordinate’ in position space by regularizing the minimum distance by a critical neighborhood size determined at the weights level and afterward correcting for the regularization bias. (This is required because for noisy spatial data and/or noncategorical reference annotations, simply taking the minimal distance between objects of certain annotations is unstable and/or not well defined.)

Specifically, TACCO first generates a matrix *n*_*A,x*_(*d*) of occurrence histograms versus spatial distance *d* for every annotation category *A* and spatial position *x*, counting fractional annotations as fractional occurrence counts. The distance $d_{A,x}^l$ where its cumulative sum over distance $N_{A,x}\left( d \right) = \mathop {\sum}\nolimits_{{{{\mathrm{d}}}}^{\prime} = 0}^{{{\mathrm{d}}}} {n_{A,x}\left( {d^{\prime} } \right)}$ will be over a certain threshold *N*_*l*_ is a robust measure for the radius of the sphere centered at *x* and containing a total of *N*_*l*_ occurrences of annotation *A*. Even for data homogeneously annotated with *A*, the minimal possible value of $d_{A,x}^l$ is however larger than 0 as the threshold *N*_*l*_ will in general be larger than *l* to have a stabilizing effect. To allow for a minimum value of 0 and obtain a measure for the distance from *x* to a significant amount of category *A*, $d_{A,x}^l$ is corrected using a fictitious homogeneous annotation category *H* and the value of *N*_*H,x*_($d_{A,x}^l$) is found, followed by solving for the distance $d_{A,x}^0$ at which *N*_*l*_ less occurrences appeared: *N*_*H,x*_($d_{A,x}^0$) = *N*_*H,x*_($d_{A,x}^l$) – *N*_*l*_. $d_{A,x}^0$ is now consistent with the regular minimum distance for noise-free categorical annotations, stable against noise and well defined for compositional annotations.

### Enrichments

TACCO supports various approaches to visualize compositional differences in the spatial structure of a sample and estimate the statistical significance of these differences/enrichments (Extended Data Fig. 3d–g). In particular, TACCO uses sample information to calculate enrichments not across observations (single cells/spatial beads, which are no independent observations and can lead to *P* value inflation), but across multiple samples, which gives meaningful and reasonable *P* values.

When there is just a single (or few) spatial sample(s) but with considerable size, different parts of that sample are treated as semi-independent biological replicates, by splitting the sample along a selection of coordinate axes to create a statistical ensemble for enrichment analysis. Increasing the number of splits to parts that cannot be regarded as independent replicates interpolates smoothly to where every observation is considered independent.

### Reporting summary

Further information on research design is available in the Nature Portfolio Reporting Summary linked to this article.

## Online content

Any methods, additional references, Nature Portfolio reporting summaries, source data, extended data, supplementary information, acknowledgements, peer review information; details of author contributions and competing interests; and statements of data and code availability are available at 10.1038/s41587-023-01657-3.

### Supplementary information

Reporting Summary

## Data Availability

The datasets analyzed during the current study are available from http://linnarssonlab.org/osmFISH/availability/, https://www.ncbi.nlm.nih.gov/geo/query/acc.cgi?acc=GSE121891, https://www.ncbi.nlm.nih.gov/geo/query/acc.cgi?acc=GSE169012, https://github.com/AllonKleinLab/paper-data/tree/master/Lineage_tracing_on_transcriptional_landscapes_links_state_to_fate_during_differentiation and https://singlecell.broadinstitute.org/single_cell/study/SCP2038.

## Code availability

TACCO is available as the open-source python package tacco, with source code freely available at https://github.com/simonwm/tacco and corresponding documentation at https://simonwm.github.io/tacco/.
