# Supplementary material for: TACCO unifies annotation transfer and decomposition of cell identities for single-cell and spatial omics
Source: Nat Biotechnol. 2023 Feb 16;41(10):1465–73. doi: 10.1038/s41587-023-01657-3 (PMC10513360; doi:10.1038/s41587-023-01657-3)
Supplement: Supplementary file 1 — Reporting Summary [file 41587_2023_1657_MOESM1_ESM.pdf]

nature research

Corresponding author(s): Mor Nitzan

Last updated by author(s): Dec 1, 2022

Reporting Summary

Nature Research wishes to improve the reproducibility of the work that we publish. This form provides structure for consistency and transparency in reporting. For further information on Nature Research policies, see our Editorial Policies and the Editorial Policy Checklist.

Statistics

For all statistical analyses, confirm that the following items are present in the figure legend, table legend, main text, or Methods section.

n/a	Confirmed	
☒	☐	The exact sample size (*n*) for each experimental group/condition, given as a discrete number and unit of measurement
☒	☐	A statement on whether measurements were taken from distinct samples or whether the same sample was measured repeatedly
☐	☒	The statistical test(s) used AND whether they are one- or two-sided<br>*Only common tests should be described solely by name; describe more complex techniques in the Methods section.*
☒	☐	A description of all covariates tested
☐	☒	A description of any assumptions or corrections, such as tests of normality and adjustment for multiple comparisons
☐	☒	A full description of the statistical parameters including central tendency (e.g. means) or other basic estimates (e.g. regression coefficient) AND variation (e.g. standard deviation) or associated estimates of uncertainty (e.g. confidence intervals)
☐	☒	For null hypothesis testing, the test statistic (e.g. *F*, *t*, *r*) with confidence intervals, effect sizes, degrees of freedom and *P* value noted<br>*Give P values as exact values whenever suitable.*
☒	☐	For Bayesian analysis, information on the choice of priors and Markov chain Monte Carlo settings
☒	☐	For hierarchical and complex designs, identification of the appropriate level for tests and full reporting of outcomes
☐	☒	Estimates of effect sizes (e.g. Cohen's *d*, Pearson's *r*), indicating how they were calculated

Our web collection on statistics for biologists contains articles on many of the points above.

Software and code

Policy information about availability of computer code

Data collection	The study does not contain experimental data collection.
Data analysis	TACCO is available as the open-source python package tacco, with source code available at https://github.com/simonwm/tacco and corresponding documentation at https://simonwm.github.io/tacco/.<br>We benchmark TACCO against publicly available packages:<br>scipy (computing NNLS, version 1.7.0),<br>NMFreg adjusted from https://github.com/tudaga/NMFreg_tutorial/blob/master/NMFreg_Tutorial_cerebellum_puck180430_6.ipynb; commit 558364d20ebf8ce98862290c3c2eef588f1faf85),<br>RCTD (version 1.2.0),<br>Waddington-OT (version 1.0.8.post2),<br>Tangram (version 4.0.4),<br>novoSpaRc (version 0.4.3),<br>SingleR (version 1.6.1),<br>scikit-learn (computing SVM, version 0.24.2),<br>Squidpy (version 1.1.1),<br>Baysor (version 0.5.2),<br>SSAM (version 1.0.2).<br>We adjusted the implementation of the RAS/Sinkhorn-Knopp matrix scaling algorithm from the package pot (version 0.7.0) to solve semi-balanced optimal transport, and a sparse variant of it for the object splitting, and combined simulation models from scsim(https://github.com/dylkot/scsim; commit 20011651341c70cbda8e41f6446380b4435693ab) and CellBender(version 0.1.0).

For manuscripts utilizing custom algorithms or software that are central to the research but not yet described in published literature, software must be made available to editors and reviewers. We strongly encourage code deposition in a community repository (e.g. GitHub). See the Nature Research guidelines for submitting code & software for further information.

April 2020

Data

Policy information about availability of data

All manuscripts must include a data availability statement. This statement should provide the following information, where applicable:

- Accession codes, unique identifiers, or web links for publicly available datasets
- A list of figures that have associated raw data
- A description of any restrictions on data availability

The datasets analyzed during the current study are available from http://linnarssonlab.org/osmFISH/availability/, https://www.ncbi.nlm.nih.gov/geo/query/acc.cgi?acc=GSE121891, https://www.ncbi.nlm.nih.gov/geo/query/acc.cgi?acc=GSE169012, https://github.com/AllonKleinLab/paper-data/tree/master/Lineage_tracing_on_transcriptional_landscapes_links_state_to_fate_during_differentiation and https://singlecell.broadinstitute.org/single_cell/study/SCP2038.

Field-specific reporting

Please select the one below that is the best fit for your research. If you are not sure, read the appropriate sections before making your selection.

☒ Life sciences ☐ Behavioural & social sciences ☐ Ecological, evolutionary & environmental sciences

For a reference copy of the document with all sections, see nature.com/documents/nr-reporting-summary-flat.pdf

Life sciences study design

All studies must disclose on these points even when the disclosure is negative.

Sample size	For all real datasets, the complete data was used -- in main text, colon Slide-seq: Fig. 2b, sematosensory osmFISH single-molecule: Fig. 2cd, hematopoiesis differentiation: Fig.3c. We used sampling over a wide range of (large) sizes in order to benchmark runtime and memory usage (Fig.4).
Data exclusions	No data were excluded
Replication	Experimental replication was not attempted and is not applicable to this study as no new data is generated.
Randomization	No datasets were generated during the current study. Random sampling was persistently used in-silico whenever relevant (e.g., in benchmarking accuracy, runtime and memory usage in Fig.4)
Blinding	No datasets were generated during the current study, and therefore blinding was not applicable.

Reporting for specific materials, systems and methods

We require information from authors about some types of materials, experimental systems and methods used in many studies. Here, indicate whether each material, system or method listed is relevant to your study. If you are not sure if a list item applies to your research, read the appropriate section before selecting a response.

Materials & experimental systems

n/a	Involved in the study	
☒	☐	Antibodies
☒	☐	Eukaryotic cell lines
☒	☐	Palaeontology and archaeology
☒	☐	Animals and other organisms
☒	☐	Human research participants
☒	☐	Clinical data
☒	☐	Dual use research of concern

Methods

n/a	Involved in the study	
☒	☐	ChIP-seq
☒	☐	Flow cytometry
☒	☐	MRI-based neuroimaging
